# Machine Learning–Assisted Analysis of the Oral Cancer Immune Microenvironment: From Single‐Cell Level to Prognostic Model Construction

**DOI:** 10.1111/jcmm.70637

**Published:** 2025-06-02

**Authors:** Ling Yang, Lijuan Guo, Yun Zhu, Zehan Zhang

**Affiliations:** ^1^ Department of Nursing Chengdu Xinhua Hospital Affiliated to North Sichuan Medical College Chengdu China; ^2^ Department of Nursing Qionglai Hospital of Traditional Chinese Medicine Chengdu China; ^3^ Department of Oral and Maxillofacial Surgery, Zhongshan Hospital Fudan University Shanghai China

**Keywords:** immune infiltration, immune microenvironment, machine learning, oral cancer, prognostic model, single‐cell sequencing

## Abstract

Oral cancer is among the most prevalent malignant tumours worldwide; prognosis can be affected by several factors, including molecular subtypes, immune microenvironment and clinical characteristics. In this study, we aimed to apply machine learning methods in conjunction with single‐cell sequencing data to characterise the immune microenvironment of oral cancer and build an immune infiltration prediction model to provide a theoretical basis for the personalised therapy and prognosis assessment of oral cancer. Clinico‐genomic data were obtained from patients with oral cancer and single‐cell sequencing was utilised to delineate the immune cell composition in the tumour microenvironment. Model construction and immune‐related gene screening were performed using machine learning algorithms such as Lasso regression, random forest and gradient boosting machine. We assessed the predictive performance of the model by cross‐validation on its training dataset and by testing the model on an independent dataset. Certain subsets of immune cells correlate with the prognosis of patients with oral cancer. C‐index (given in supplementary) yielded a good discrimination ability (C‐index > 0.75) in the training set and validation set. Moreover, the model‐identified immune‐related genes presented remarkable expression differences in the two different risk groups and played important roles in the response to immune therapy. By exploring the complexity of the oral cancer immune microenvironment with machine learning techniques, in this study, we build a reliable prognostic model based on immune infiltration. The model could be applied in clinical practice to personalisation treatment decision‐making and prognosis evaluation.

## 1 Introduction

Oral cancer, especially oral squamous cell carcinoma (OSCC), is one of the most prevalent forms of head and neck cancers globally, with a high incidence and mortality rate. It is a major burden to patients, their families and public health systems [1, 2, 3]. Think of the pathogenesis of oral cancer, which is very complex and involved. Poor overall survival rates are attributed to challenges, including the difficulty of early diagnosis, resistance to treatment and high recurrence rates of diseases [4, 5, 6, 7]. As a result, a comprehensive understanding of the biological attributes and molecular mechanisms of oral cancer is important for early diagnosis, treatment and prediction of prognosis.

The tumour microenvironment, particularly immune cell infiltration, has been increasingly recognised as a key contributor to a tumour's initiation, progression, metastasis and response to therapy (PD‐L1; Programmed cell death‐ligand 1). Leukocyte infiltration, including T cells, B cells, macrophages and dendritic cells in oral cancer, correlates to patient prognosis. These immune cells are not only involved in tumour immune surveillance but can also interact with cancer cells and affect the biological behaviour of tumours. High heterogeneity and complexity of immune infiltration will bring new opportunities and challenges for oral cancer treatment and prognosis [8, 9, 10]. The functional status of immune cells in the tumour microenvironment and its use in guiding immunotherapy development have become a research hotspot with the rise of immunotherapy.

Machine learning, as a powerful data analysis tool, has great potential in cancer research in the era of big data. Machine learning algorithms can recognise patterns and correlations in complex biomedical data, aiding researchers in identifying new biomarkers, predicting disease course and response to therapies and developing prognostic models [11, 12, 13]. Machine learning helps in retrieving an extensive tumour microenvironment and customising the treatment; however, the application of machine learning in the oral cancer setting is only in its infancy. Large‐scale genomic, transcriptomic, proteomic and clinical studies can be analysed and integrated using machine learning models, which lead to the discovery of the molecular subtypes and biological characteristics of tumours, bringing new breakthroughs in precision medicine for oral cancer.

In this study, we will use a variety of machine learning methods, including Random Forest, Gradient Boosting Machine and Lasso Regression, to process large single‐cell sequencing datasets. The use of these algorithms can allow us to characterise immune infiltration–related gene expression patterns and construct predictive models. We have, in fact, hypothetically trained the models through cross‐validation and independent dataset testing such that we examine the predictive performance and generalisation ability of the models. We will also assess the discrimination ability and predictive accuracy of the models using survival analysis and the C‐index respectively. This study aims to identify new characteristics of immune cell infiltration in oral cancer and establish a highly predictive prognostic model. These findings will offer new targets for precision treatment of oral cancer and provide a scientific basis for personalised treatment and prognosis assessment of patients. Your response will create a one‐of‐a‐kind combination of words.

## 2 Methods

### 2.1 Data Acquisition

We collected comprehensive RNA expression data and clinical profiles for oral cancer (OC) patients from The Cancer Genome Atlas (TCGA) and the Gene Expression Omnibus (GEO) databases [14]. Moreover, single‐cell RNA sequencing (scRNA‐seq) data were retrieved from the GSE103322 dataset.

### 2.2 Identification of OC‐Associated Genes

Employing R software, we conducted differential expression analysis on the gene expression profiles between OC tumour samples and their corresponding normal tissue samples, resulting in the identification of 276 differentially expressed genes associated with OC. This process involved rigorous statistical testing to pinpoint genes with significant expression alterations in OC. We set a log fold change (logFC) threshold of 1 to ensure a minimum twofold difference in gene expression and utilised a false discovery rate (FDR) threshold of 0.05 to manage the false positive rate, thereby ensuring statistical significance and addressing multiple testing challenges. Subsequently, we performed univariate Cox regression analysis on the differentially expressed OC‐associated genes to evaluate the correlation between gene expression levels and patient prognosis. Additionally, we carried out correlation analysis between gene expression and clinical data to uncover potential prognostic biomarkers. Researchers also explored the link between autophagy‐related genes and immune cell infiltration in OC, which may elucidate intricate interactions in OC pathogenesis. To address the identification of the 276 differentially expressed genes (DEGs), we employed a rigorous statistical approach. We utilised the negative binomial test, commonly applied in transcriptomic analyses, to compare gene expression levels between tumour and normal samples. This method effectively handles the discrete and heterogeneous nature of gene expression data, providing log2 fold changes (L2FC) and corresponding *p* values for each gene. To control the false discovery rate (FDR), we applied the Benjamini–Hochberg correction, which adjusts *p* values to account for multiple testing. Genes were selected based on an L2FC threshold of 1 (indicating at least a 2‐fold change in expression) and an FDR‐adjusted *p* value threshold of 0.05 to ensure statistical significance. Additionally, we will provide a comprehensive list of these DEGs, including their names, L2FC values original *p* values and adjusted *p* values, either in the main text or supporting informations. This detailed information will enhance transparency and reproducibility, allowing further exploration of these genes' biological roles in oral cancer.

### 2.3 Consensus Clustering Methodology

The ‘ConsensusClusterPlus’ R package was utilised for unsupervised analysis to ascertain the optimal number of clusters and their constituents based on stability evidence. This methodology involves multiple iterations of clustering on 80% of the data to assess cluster stability and reliability. Within the consensus clustering framework, we applied the k‐means algorithm, an iterative clustering technique that commences with the arbitrary selection of K objects as initial cluster centroids and subsequently assigns each data point to the nearest cluster based on distance calculations. We generated item consensus plots and cluster consensus plots to appraise the consistency level of each gene and cluster, which are instrumental in determining the optimal cluster number and member stability. The ‘ConsensusClusterPlus’ package facilitated the creation of a consensus matrix and consensus tree, enabling us to decipher the clustering structure of the data and identify two principal clusters: ‘OC‐related’ and ‘non‐OC‐related’. To compare survival rates between these clusters, we employed Kaplan–Meier survival analysis, a nonparametric method for summarising survival probabilities. This analysis permitted us to evaluate the correlation between distinct clusters and patient prognosis. Prior to consensus clustering, data preprocessing was conducted, including L2 normalisation to mitigate batch effects across samples. For enhanced visualisation of clustering outcomes, we potentially employed the t‐SNE algorithm, a dimensionality reduction technique adept at rendering high‐dimensional data [15, 16, 17].

### 2.4 Machine Learning Approaches, Model Development and Validation

To develop and refine machine learning models, we segregated the OC dataset into two distinct cohorts (TCGA and GEO) to assess the models' generalisation capabilities across various data sources. We trained the models using 10 algorithms and 101 algorithmic combinations, encompassing random survival forests (RSF), elastic net (Enet), Lasso, ridge regression, stepwise Cox, CoxBoost, partial least squares regression for Cox models (plsRcox), super principal components (SuperPC), gradient boosting machines (GBM) and survival support vector machines (survival‐SVM). These algorithms excel in high‐dimensional data scenarios and are renowned for feature selection, particularly Lasso. Model selection was guided by the maximum average Harrel's concordance index (C‐index) derived from both datasets. The C‐index, a statistical metric, quantifies predictive accuracy on a scale from [0, 1], with values closer to 1 indicating superior model performance. The risk score for each sample was calculated using the formula: ‘Risk score = Σ (coefficient × expression value)’, allowing for patient stratification based on risk levels. For data visualisation, including Sankey diagrams to illustrate the interconnections between the two risk clusters, we leveraged the R package ‘ggplot2’. Two‐dimensional Sankey diagrams visualise the contribution of various risks to outcomes. The dataset was partitioned into overall, TCGA‐specific and GEO‐specific subsets for Kaplan–Meier survival analysis, complemented by 10‐fold cross‐validation to generate ROC curves and decision curve analysis (DCA) to substantiate model robustness [18, 19, 20, 21]. The Kaplan–Meier method, a nonparametric approach estimating the survival function and the receiver operating characteristic, ROC curves, which assess the model's ability to distinguish between survival outcomes, were employed. In model development, we adopted cross‐validation techniques, such as k‐fold cross‐validation, to test model accuracy, with k representing the number of data splits. One set is reserved as the test set, while the remainder are used as the training set, a process repeated k times to mitigate variance in model evaluation and yield a more reliable performance estimate.

### 2.5 Functional Enrichment and Immune Infiltration Analysis

Gene ontology (GO) classification and KEGG pathway enrichment analysis were conducted to investigate the roles of differentially expressed genes (DEGs) in OC and non‐OC groups, with an FDR threshold of < 0.05 for statistical significance. The CIBERSORT and ESTIMATE algorithms in R were applied to discern variations between cohorts [22, 23, 24].

### 2.6 Single‐Cell Data Validation

The ‘Seurat’ R package was employed for data preprocessing, initially eliminating cells with fewer than 200 features and those with mitochondrial gene percentages exceeding 20% to ensure data integrity. Standard logarithmic normalisation (LogNormalisation) was applied to the data, with the scaling factor L set to 1, to mitigate biases and noise and render the data more amenable to subsequent analysis. The ‘LogNormalization’ method normalised feature expression levels of each cell by total expression and converted to logarithmic form to reduce batch effects across samples. Subsequently, L2 normalisation was applied to derive an M × *N* data matrix, with each element representing the normalised expression values. Unsupervised clustering analysis grouped cells into distinct clusters based on gene expression similarity, revealing inherent cellular heterogeneity without prior knowledge. Principal component analysis (PCA) and t‐SNE were utilised for data dimension reduction and visualisation [25, 26]. PCA condenses data dimensionality, while t‐SNE projects high‐dimensional data clustering structure into two‐dimensional space. The ‘SingleR’ package compared the scRNA‐seq data with known reference datasets to identify and annotate cell types within each cluster, providing an unbiased cell type identification approach independent of known marker genes. The ‘FindAllMarkers’ package identified marker genes with significant differential expression across various cell types, offering a range of statistical testing methods, including *t*‐tests and negative binomial generalised linear models, to discern differentially expressed genes between distinct cell populations.

## 3 Results

### 3.1 The Impact of Different Factors on Overall Survival in Oral Cancer Patients

**FIGURE 1:**
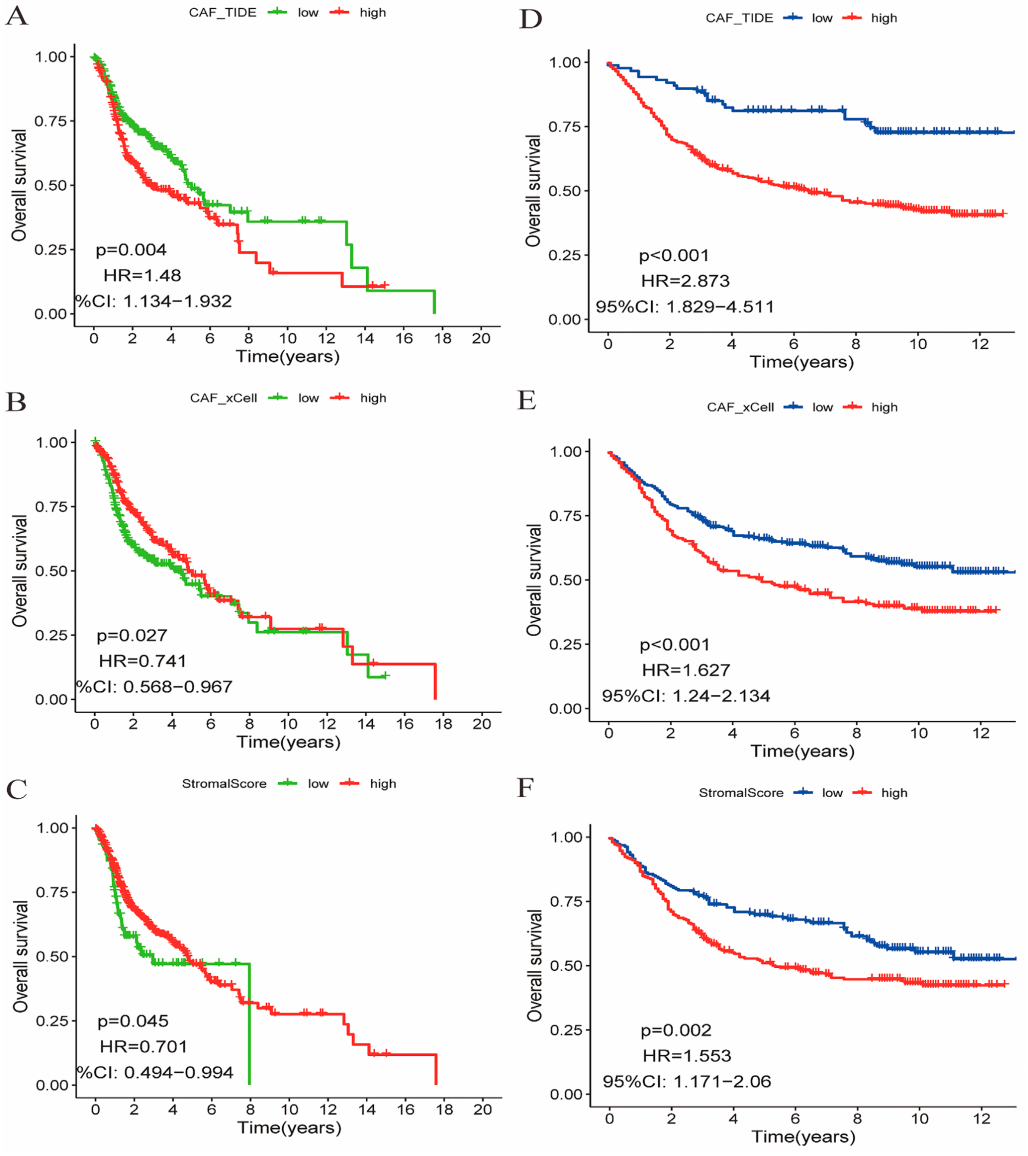
The impact of different factors on overall survival in oral cancer patients. A‐C: Kaplan–Meier survival curves showing overall survival based on low (green) and high (red) levels of CAF_TIDE, CAF_xCell and StromalScore. (A) Low CAF_TIDE levels are linked to better survival (*p* = 0.004, HR = 1.48). (B) Low CAF_xCell levels improve survival (*p* = 0.027, HR = 0.741). (C) Low StromalScore is associated with better survival (*p* = 0.045, HR = 0.701). D‐F: Survival curves with high (blue) and low (red) groups, confirming previous trends. (D) High CAF_TIDE levels indicate poorer survival (*p* < 0.001, HR = 2.873). (E) High CAF_xCell levels relate to worse survival (*p* < 0.001, HR = 1.627). (F) High StromalScore correlates with reduced survival (*p* = 0.002, HR = 1.553).

Figure 1A shows that the high CAF_TIDE group has lower survival rates, with a statistically significant *p* = 0.004 and HR = 1.48 (95% CI = [1.134, 1.932]). Figure 1B demonstrates that the high CAF_xCell group also experiences lower survival rates, with *p* = 0.027 and HR = 0.741 (95% CI = [0.568, 0.967]). Figure 1C indicates that the high StromalScore group has reduced survival rates, with *p* = 0.045 and HR = 0.701 (95% CI = [0.494, 0.994]). Figure 1D highlights that the high CAF_TIDE group shows significantly lower survival compared to the low group, with *p* < 0.001 and HR = 2.873 (95% CI = [1.829, 4.511]). Figure 1E reveals that the high CAF_xCell group has lower survival rates, with *p* < 0.001 and HR = 1.627 (95% CI = [1.24, 2.134]). Figure 1F shows that the high StromalScore group experiences decreased survival, with *p* = 0.002 and HR = 1.553 (95% CI = [1.171, 2.06]). Overall, higher levels of CAF_TIDE, CAF_xCell and StromalScore are associated with poorer survival outcomes in oral cancer patients.

### 3.2 Module–Trait Relationships, Gene Significance and Functional Enrichment

**FIGURE 2:**
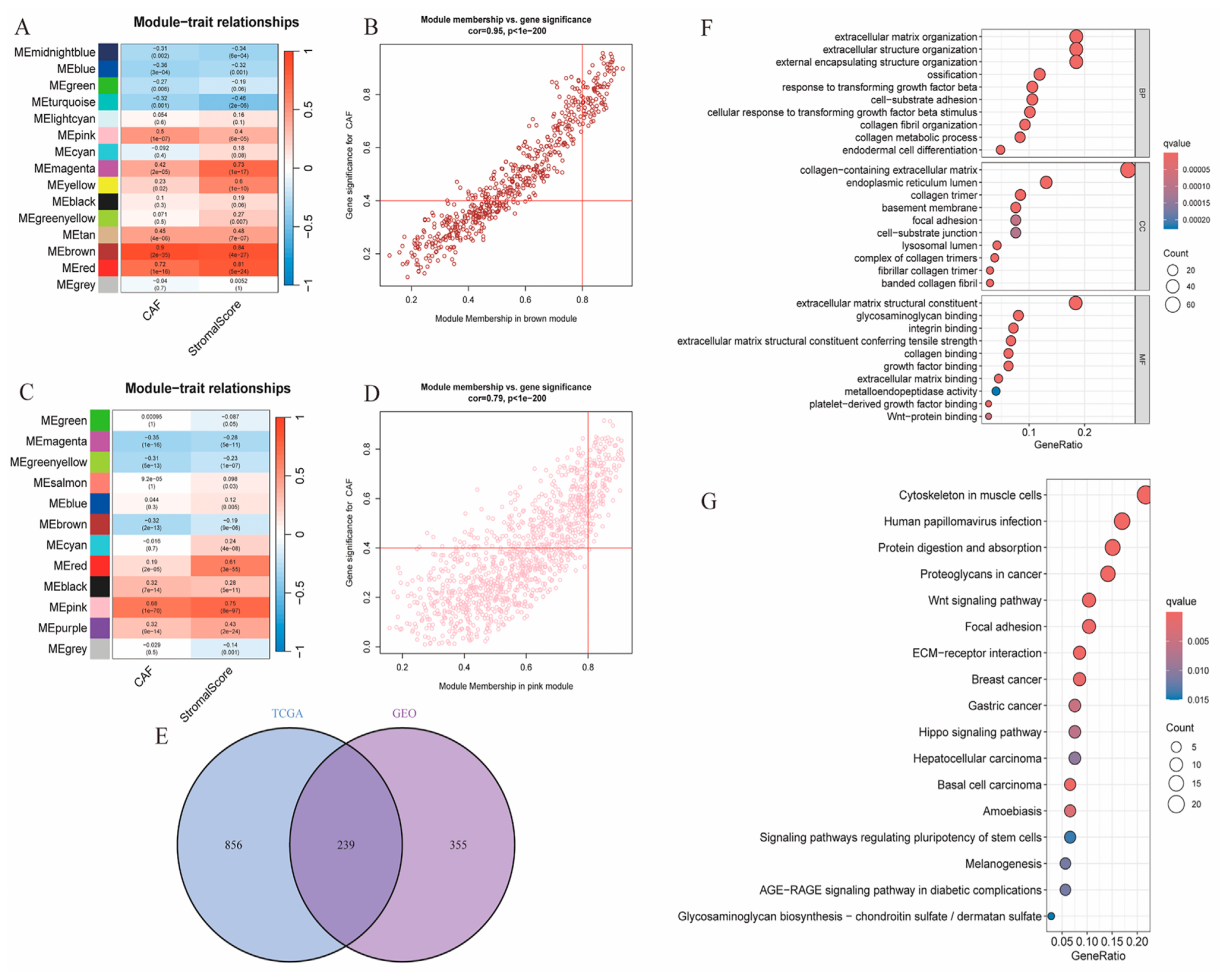
Module–trait relationships, gene significance and functional enrichment. (A) Heatmap showing module–trait relationships, indicating correlations between gene modules and traits. (B) Scatter plot of module membership versus gene significance for a specific module, showing a strong positive correlation. (C) Another heatmap of module–trait relationships for a different dataset. (D) Scatter plot similar to Panel B, but with a weaker correlation. (E) Venn diagram displaying the overlap of genes between two datasets (TCGA and GEO). (F) Dot plot of enriched biological processes, highlighting significant pathways related to extracellular matrix organisation. (G) Dot plot showing enriched pathways, focusing on cancer‐related and signalling pathways.

Figure 2A,C Module–Trait Relationships: Heatmaps display the correlation between gene modules and specific traits. Different colours indicate the strength and direction of these correlations. For instance, certain modules show strong positive or negative correlations with traits like OS (overall survival) and T (tumour). Figure 2B,D: These plots illustrate the relationship between module membership and gene significance. A trend is visible, suggesting that genes highly significant for a trait are also central to the corresponding module. Figure 2E: This diagram shows the overlap between two datasets, indicating shared and unique elements. The intersection highlights common genes or features between the datasets. Figure 2F,G: Dot plots display enriched biological processes and pathways. The size of the dots represents the count of genes involved, while the colour gradient indicates the significance level (*p* value). Key pathways like ‘extracellular matrix organization’ and ‘immune response’ are prominently enriched, suggesting their roles in the study's context.

### 3.3 Analysis of Model Performance and Gene Interactions

**FIGURE 3:**
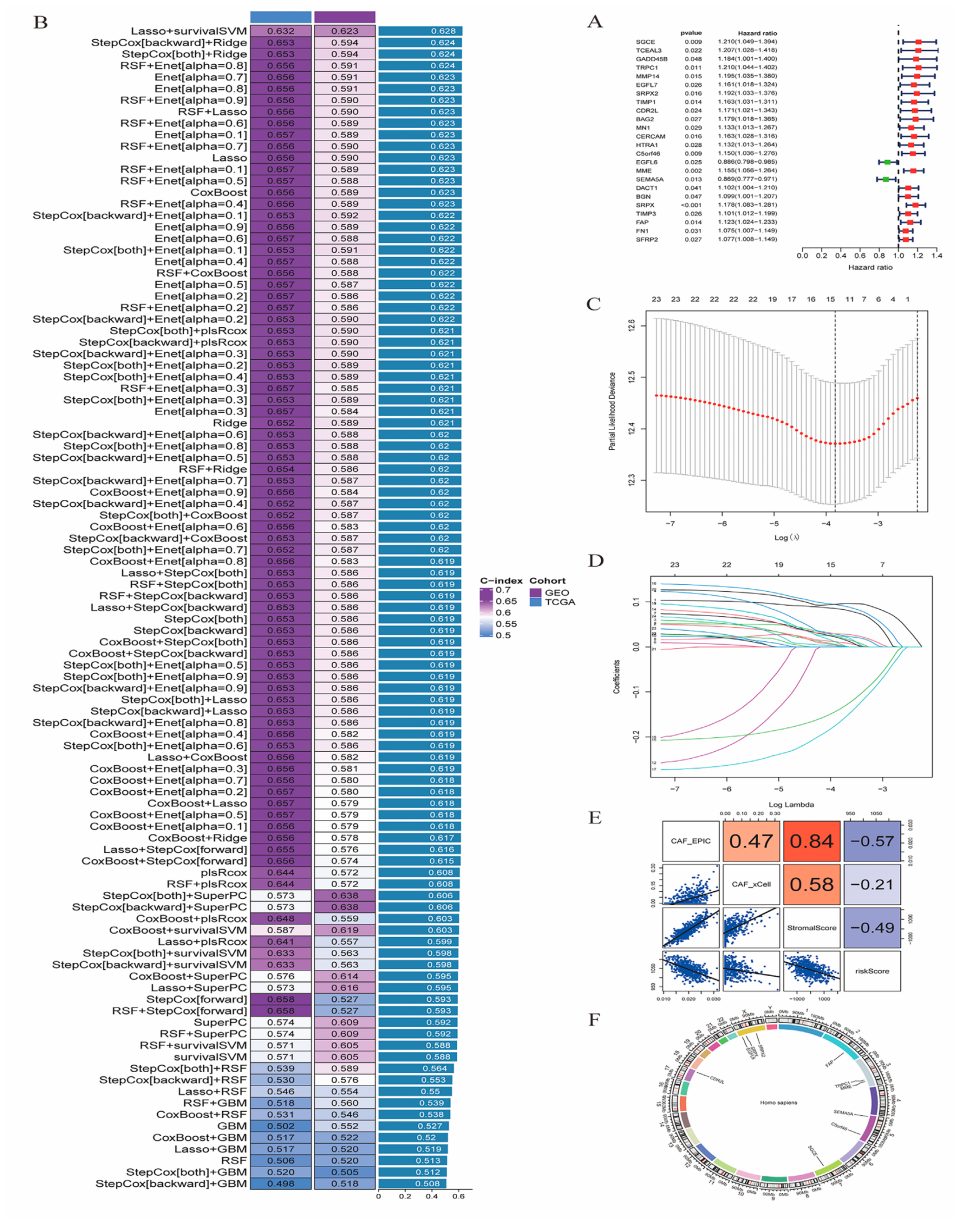
Analysis of model performance and gene interactions. (A0 Forest plot showing hazard ratios and confidence intervals for various factors. (B) Heatmap of C‐index values for different models across cohorts, with colour indicating performance. (C) Plot of cross‐validation error rates for selecting model parameters. (D) Coefficient paths for variables in a regularisation model. (E) Correlation matrix with scatter plots and correlation coefficients. (F) Circular plot illustrating interactions among key genes or pathways.

Figure 3A: A forest plot displays the hazard ratios of various genes, indicating their impact on survival. Each gene's significance is represented by the position and length of the lines, with dots marking the hazard ratio values. Figure 3B: A heatmap shows the performance of different predictive models across two cohorts (GEO and TCGA). The C‐index values, represented by colour intensity, indicate the models' predictive accuracy, with higher values reflecting better performance. Figure 3C: A plot of partial likelihood deviance versus log(lambda) from a Lasso regression identifies the optimal model complexity. The red dotted line marks the lambda value with the smallest deviance, suggesting the best balance between model fit and complexity. Figure 3D: A coefficient path plot illustrates how gene coefficients change with varying levels of regularisation in the Lasso model. This helps identify which genes remain significant predictors across different model complexities. Figure 3E: A correlation matrix visualises the relationships between key variables, with colour intensity and values indicating the strength and direction of correlations. Strong positive and negative correlations are highlighted, providing insights into gene interactions. Figure 3F: A circular plot (circos plot) represents gene interactions and their chromosomal locations. Arcs and lines connect interacting genes, illustrating the network of relationships and their genomic context.

### 3.4 Analysis of Risk Factors and Survival Outcomes

**FIGURE 4:**
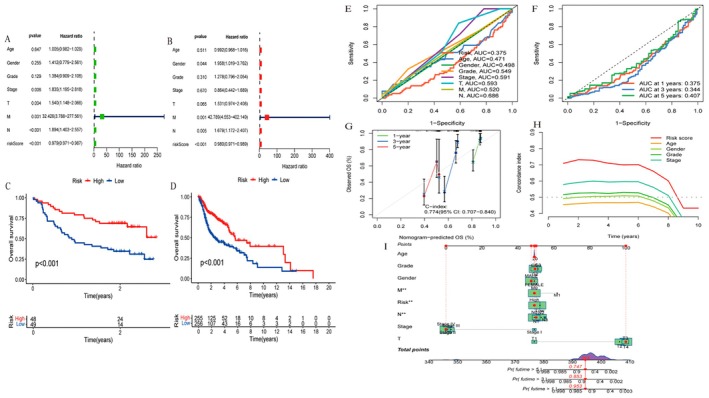
Analysis of model performance and gene interactions. (A) Forest plot showing hazard ratios for clinical factors and risk scores, highlighting significant predictors. (B) Forest plot for another dataset, emphasising key risk factors with hazard ratios. (C) Kaplan–Meier survival curve comparing high and low‐risk groups over 2 years, showing significant differences (*p* < 0.001). (D) Kaplan–Meier survival curve for high and low‐risk groups over 20 years, also showing significant differences (*p* < 0.001). (E) ROC curves showing AUC values for different factors, indicating predictive accuracy. (F) ROC curves for 1‐, 3‐ and 5‐year predictions, with corresponding AUC values. (G) Calibration plot for nomogram predictions at 1, 3 and 5 years, with a C‐index of 0.774. (H) Concordance index over time for various predictors, showing risk score as the strongest predictor. (I) Nomogram for predicting overall survival, integrating multiple clinical factors.

Figure 4A: A forest plot shows the hazard ratios for various clinical factors, including age, gender, grade, stage and risk score, in the TCGA cohort. Significant factors are highlighted, with risk score having a strong association with survival (*p* < 0.001). Figure 4B: Similar to Figure 4A, this forest plot displays hazard ratios for the GEO cohort. The risk score remains a significant predictor of survival, with a notable hazard ratio (*p* < 0.001). Figure 4C: A Kaplan–Meier survival curve for the TCGA cohort compares overall survival between high‐risk and low‐risk groups. The high‐risk group (blue line) shows significantly lower survival rates than the low‐risk group (red line), with a *p* value < 0.001. Figure 4D: This Kaplan–Meier curve for the GEO cohort also compares survival between high‐risk and low‐risk groups. Consistent with the TCGA results, the high‐risk group has poorer survival outcomes, with a *p* value < 0.001. Figure 4E: ROC curves for various clinical factors, including risk score, age, gender, grade, stage and others, show their ability to predict outcomes. The AUC values indicate the predictive power, with stage and N (node involvement) having relatively higher AUCs. Figure 4F: ROC curves for 1‐year, 3‐year and 5‐year survival predictions demonstrate the model's performance over time. The AUC values suggest moderate predictive accuracy for these time points. Figure 4G: A calibration plot compares observed overall survival (OS) with nomogram‐predicted OS at 1, 3 and 5 years. The C‐index of 0.774 (95% CI: 0.707–0.840) indicates good predictive accuracy of the nomogram. Figure 4H: A time‐dependent concordance index plot shows the performance of different factors over time. The risk score consistently maintains higher predictive accuracy compared to other factors. Figure 4I: A nomogram integrates various factors like age, gender, grade, stage and risk score to predict survival probabilities. It provides a visual tool for estimating individual patient outcomes based on total points accumulated from each factor.

### 3.5 Evaluation of the Predictive Performance of Risk Factors and Clinical Characteristics

**FIGURE 5:**
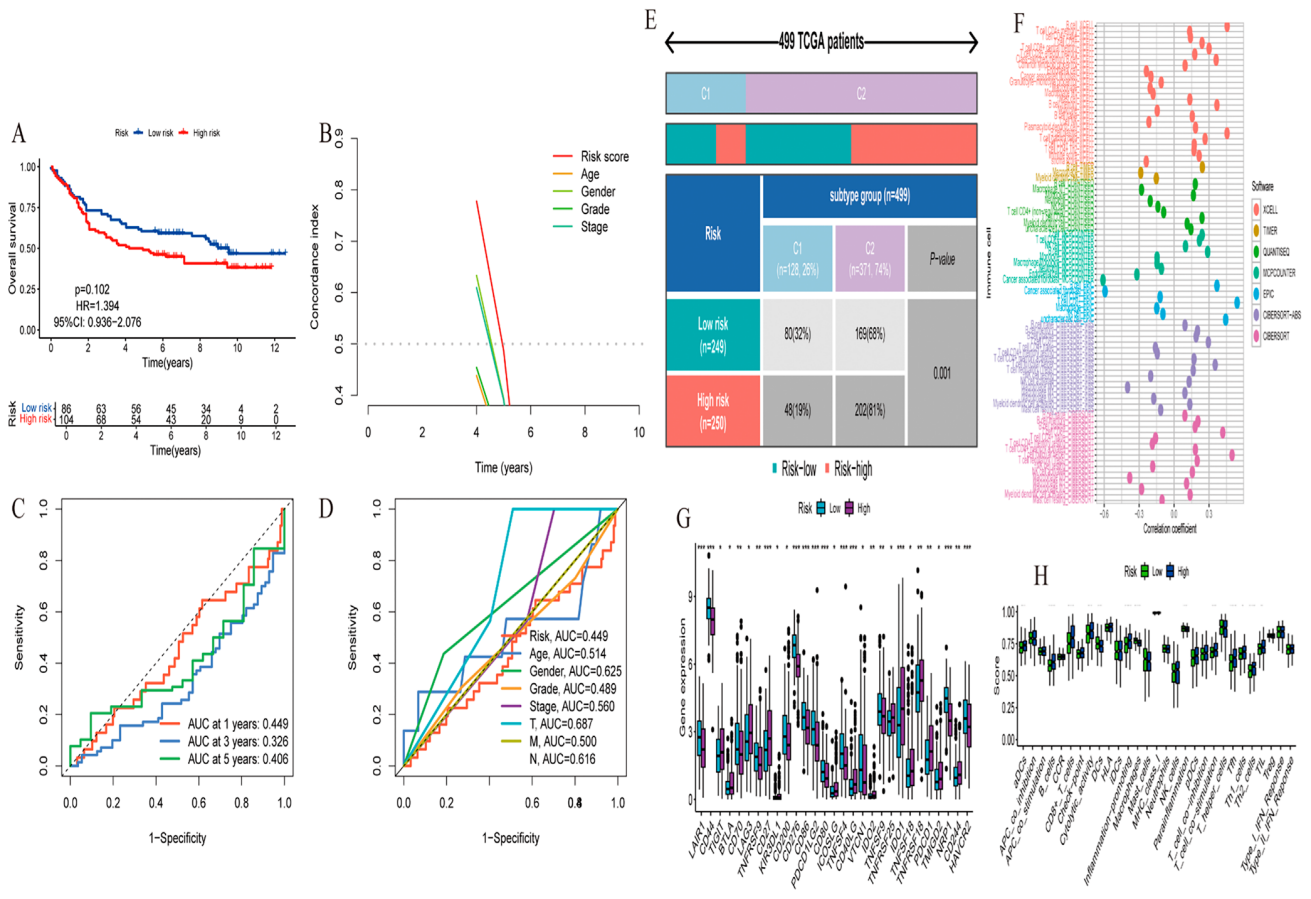
Evaluation of the predictive performance of risk factors and clinical characteristics. (A) Kaplan–Meier survival curve comparing low‐ and high‐risk groups, showing no significant difference (*p* = 0.102). (B) Concordance index over time for different predictors, highlighting the risk score. (C) ROC curves for 1, 3 and 5‐year predictions, with AUC values indicating moderate accuracy. (D) ROC curves for various clinical factors, showing their AUC values and predictive performance. (E) Patient distribution across risk and subtype groups, showing significant differences (*p* = 0.001). (F) Dot plot of correlation coefficients for various immune cell types across different methods. (G) Box plots comparing gene expression between low‐ and high‐risk groups. (H) Box plots of immune scores for low‐ and high‐risk groups.

Figure 5A The Kaplan–Meier survival curve compares overall survival between high‐risk and low‐risk groups. Although the high‐risk group (red line) shows a trend towards lower survival, the difference is not statistically significant (*p* = 0.102). The hazard ratio is 1.394, with a 95% confidence interval of 0.936–2.076. Figure 5B: A time‐dependent concordance index plot assesses the predictive accuracy of various factors over time. The risk score, age, gender, grade and stage are compared, with the risk score showing a decline in predictive power over time. Figure 5C: ROC curves for 1‐year, 3‐year and 5‐year survival predictions display the model's performance at these intervals. The AUC values are 0.449, 0.325 and 0.346, indicating limited predictive accuracy. Figure 5D: ROC curves compare the predictive power of the risk score with other clinical factors, such as age, gender, grade, stage, T (tumour size) and N (node involvement). The AUC values suggest that node involvement (N) has the highest predictive accuracy (AUC = 0.816), while the risk score has a lower AUC of 0.449. Figure 5E: A stratification plot categorises 499 TCGA patients into subtypes C1, C2 and C3. The distribution of high‐risk and low‐risk patients across these subtypes is shown, with a significant association (*p* = 0.001). The high‐risk group is predominantly in subtype C3. Figure 5F: A dot plot illustrates the correlation of various immune cell types with risk scores across different software tools. Each dot represents a correlation coefficient, with different colours indicating the software used (e.g., TIMER, CIBERSORT, etc.). Figure 5G: A box plot compares the expression of immune checkpoint genes between high‐risk and low‐risk groups. Significant differences are noted in several genes, suggesting variations in immune response based on risk level. Figure 5H: Another box plot assesses the expression of immune‐related signatures between the two risk groups. The data suggest that high‐risk patients may have distinct immune profiles compared to low‐risk patients.

### 3.6 The Relationship Between Risk Groups, Immune Response and Microenvironment Scores

**FIGURE 6:**
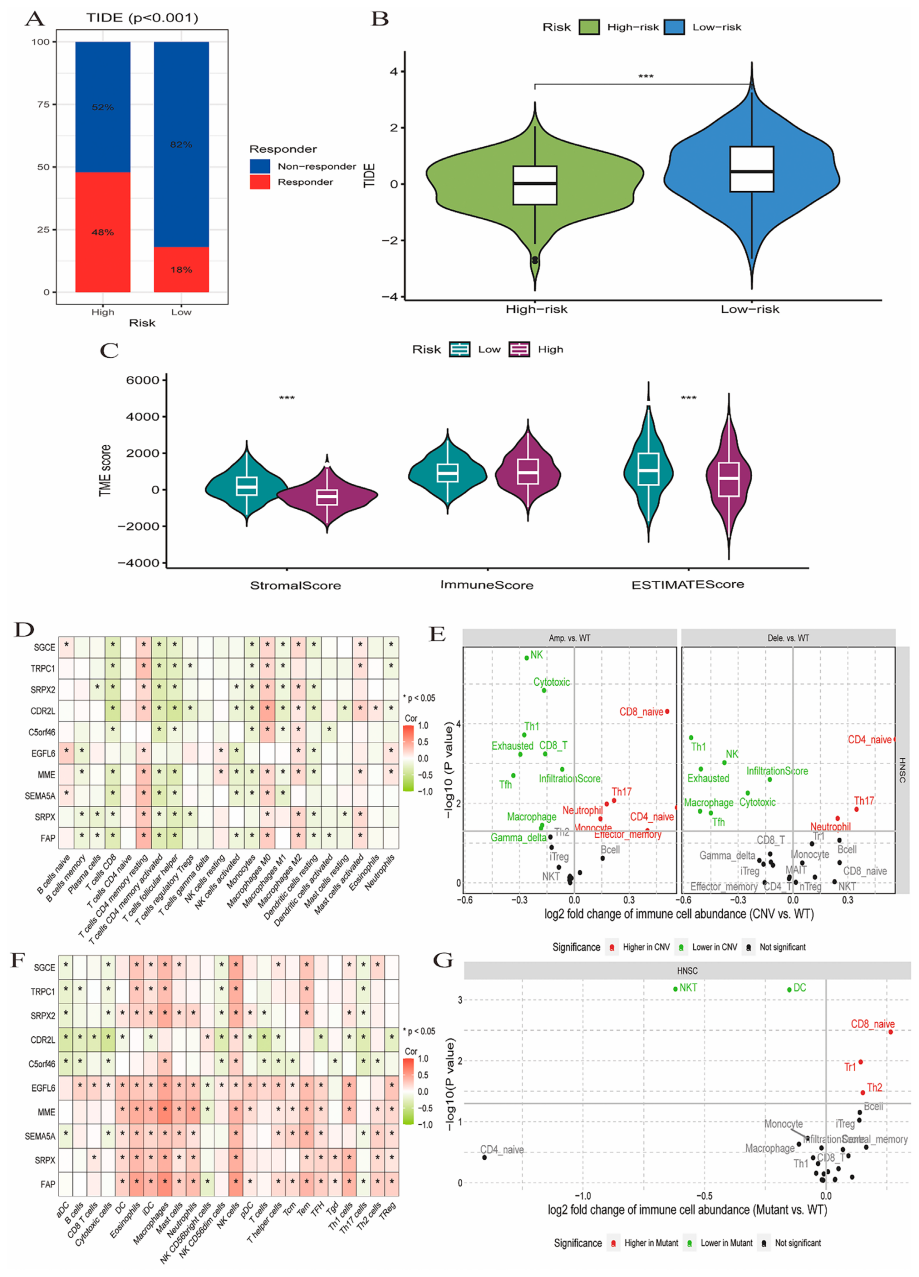
The relationship between risk groups, immune response and microenvironment scores. (A) Bar chart showing responder rates for high and low‐risk groups, with significant differences (*p* < 0.001). (B) Violin plot comparing TIDE scores between high‐risk and low‐risk groups, showing a significant difference. (C) Violin plots for StromalScore, ImmuneScore and ESTIMATEScore, comparing low‐ and high‐risk groups, with significant differences. (D) Heatmap showing correlations between genes and immune cells, with varying significance levels. (E) Another heatmap highlighting different gene‐immune cell correlations. (F) Scatter plots of immune cell abundance changes, comparing CNV with WT, indicating significant differences. (G) Scatter plot of immune cell abundance changes, comparing Mutant with WT, marking significant differences.

**FIGURE 7:**
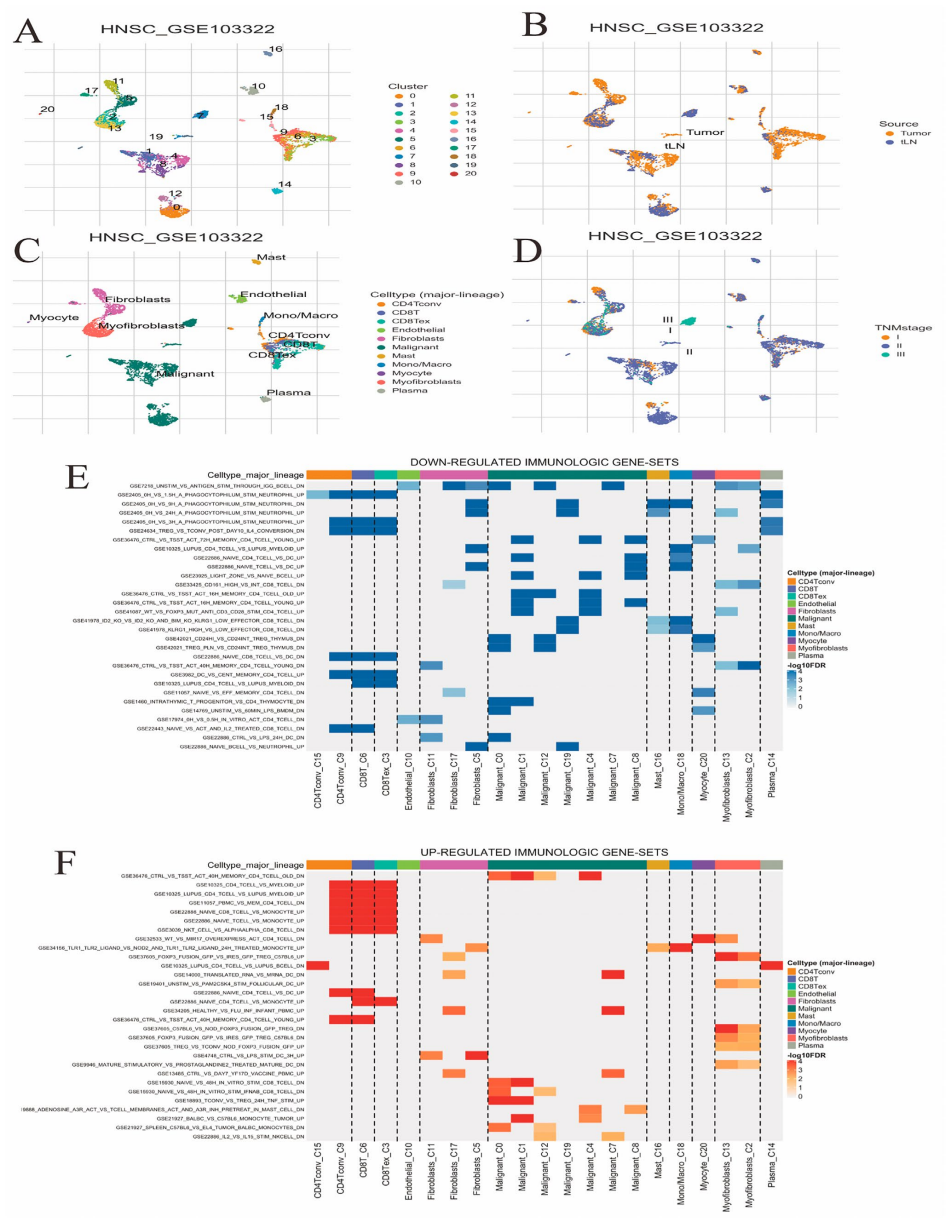
Analysis of oral cancer from the GSE103322 dataset. (A) Clustering of cells into distinct groups. (B) Cells categorised by source: Tumour versus lymph node (LN). (C) Cell types identified, including fibroblasts and immune cells. (D) Cells grouped by TNM stages I, II and III. (E) Heatmap of downregulated immunologic gene sets across different cell types. (F) Heatmap of upregulated immunologic gene sets across various cell types.

Figure 6A: A bar plot displays the TIDE (tumour immune dysfunction and exclusion) analysis results, showing the proportion of responders and nonresponders to immunotherapy in high‐risk and low‐risk groups. The high‐risk group has a lower percentage of responders (18%) compared to the low‐risk group (45%), with a significant difference (*p* < 0.001). Figure 6B: A violin plot compares TIDE scores between high‐risk and low‐risk groups. The high‐risk group has higher TIDE scores, indicating more immune dysfunction and exclusion, which is statistically significant (***p < 0.001). Figure 6C: Violin plots show the distribution of StromalScore, ImmuneScore and ESTIMATEScore between high‐risk and low‐risk groups. The low‐risk group generally has higher scores in all categories, suggesting a more favourable tumour microenvironment. The differences are statistically significant (***p < 0.001). Figure 6D: A heatmap shows the correlation between immune checkpoint genes and various immune cell types in the high‐risk group. Positive correlations are indicated in green, and negative correlations in red. Significant correlations are marked, highlighting key interactions. Figure 6E: This heatmap displays correlations in the low‐risk group. Differences in correlation patterns between high‐risk and low‐risk groups suggest variations in immune cell interactions. Figure 7F: Scatter plots illustrate the log2 fold change of immune cell abundance between CNV (Copy Number Variation) and wild‐type (WT) groups. Significant changes are highlighted, with some immune cells being more abundant in CNV or WT groups. Figure 6G: A scatter plot shows the log2 fold change of immune cell abundance between mutant and WT groups. Immune cells with significant changes in abundance are labelled, indicating how mutations influence immune cell presence.

### 3.7 Immune Cell Interactions and Gene Expression Correlations

Figure 7A: A UMAP plot shows the clustering of cells into distinct groups, labelled by cluster numbers. Each colour represents a different cluster, indicating the heterogeneity within the dataset. Figure 7B: This UMAP plot differentiates cells based on their source: tumour tissue or lymph nodes (LN). Tumour‐derived cells and LN‐derived cells are marked in distinct colours, showing the spatial distribution of cells from different origins. Figure 7C: A UMAP plot categorises cells by major cell types, such as CD8+ T cells, fibroblasts, endothelial cells and others. Each cell type is represented by a different colour, illustrating the diverse cell populations present in the sample. Figure 7D: This UMAP plot highlights cells based on TNM staging (Tumour, Node, Metastasis), with different colours representing different stages. This visualisation helps to understand how cellular composition varies with disease progression. Figure 7E: This heatmap shows downregulated immunologic gene sets. Each row represents a gene set, and columns represent different cell types, such as CD8+ T cells, fibroblasts and macrophages. Shades of blue indicate the degree of downregulation, with darker shades representing stronger downregulation. The distribution across cell types suggests specific pathways that may be suppressed in certain immune cells. Figure 7F: This heatmap displays upregulated immunologic gene sets. Similar to Panel A, each row corresponds to a gene set, and columns represent cell types. Shades of red and orange indicate the level of upregulation, with darker shades showing stronger upregulation. This highlights pathways that are more active in certain cell populations.

### 3.8 The Expression Patterns of Specific Genes in Oral Cancer at the Single‐Cell Level

**FIGURE 8:**
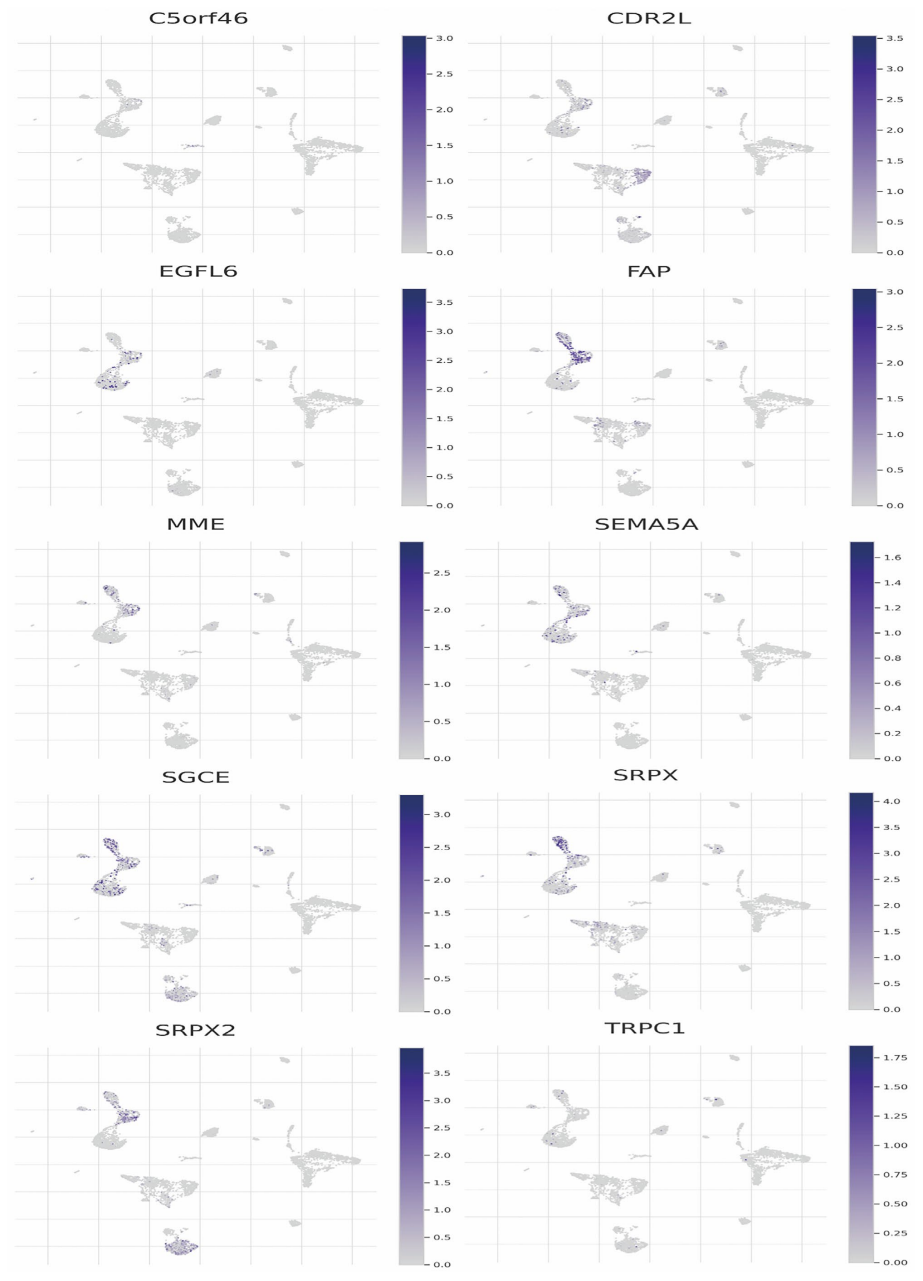
The expression patterns of specific genes in oral cancer at the single‐cell level. Expression levels of various genes (e.g., C5orf46, CDR2L, EGFL6) are shown across cell clusters, with intensity indicated by colour gradients.

Each plot corresponds to a different gene, such as C5orf46, CDR2L, EGFL6, FAP, MME, SEMA5A, SGCE, SRPX, SRPX2 and TRPC1. The intensity of blue in each plot indicates the level of gene expression, with darker shades representing higher expression levels. The spatial distribution of expression across the UMAP plots suggests the presence of these genes in specific cell clusters, highlighting their potential roles in different cell types within the tumour microenvironment (Figure 8).

## 4 Discussion

The recent advancements in genomic technologies have revolutionised our understanding of oral cancer (OC), providing insights into its molecular underpinnings and paving the way for precision medicine. This discussion focuses on a comprehensive genomic analysis and machine learning‐driven approach to identify OC‐associated genes, understand immune cell infiltration and develop prognostic models that could potentially transform OC management.

In cancer research, survival analysis is fundamental, as every effort aims to improve patient survival time or reduce the likelihood of recurrence. The application of machine learning in this field includes empirical comparisons of statistical and machine learning methods on medical datasets for different types of cancer. The performance of these techniques across various settings highlights the potential of machine learning in cancer survival analysis. Training computers with data has been highly effective in predicting various types of cancer, including breast, brain, lung, liver and prostate cancer [27, 28, 29]. Machine learning models can accurately predict which treatment options are most suitable for each patient based on their molecular, genetic and tumour characteristics. A study used machine learning techniques to classify cancer‐related data and generate diagnoses for breast cancer. This study examined different classification techniques and applied them to specific feature subsets, including Support Vector Machine (SVM) classifiers, Probabilistic Neural Networks and K‐Nearest Neighbours. The SVM classifier model showed the highest overall accuracy in breast cancer diagnosis [30, 31, 32, 33]. Age and gender are important factors that can significantly influence the immune microenvironment and consequently affect prognostic predictions in oral cancer. The current study has not thoroughly examined these demographic variables as potential modifiers of immune infiltration patterns and their impact on model performance. Similarly, biological sex differences in immune responses are well documented, with females generally demonstrating more robust immune activation than males. Sex hormones like oestrogen and testosterone directly modulate immune cell function and may influence tumour–immune interactions in oral cancer. Recent literature has shown that male and female cancer patients can respond differently to immunotherapies, suggesting that sex‐specific immune signatures might exist.

The study leverages large‐scale transcriptomic data from OC patients, sourced from reputable databases such as The Cancer Genome Atlas (TCGA) and Gene Expression Omnibus (GEO). Single‐cell RNA sequencing (scRNA‐seq) data from the GSE103322 dataset further enrich this analysis, offering a granular view of cellular heterogeneity in OC. The identification of 276 differentially expressed OC‐related genes through rigorous statistical testing and Cox regression analysis is a significant step forward in understanding the molecular signatures of OC.

The application of the ‘ConsensusClusterPlus’ R package for unsupervised analysis is a robust method to determine the number of clusters and their membership based on stability evidence. This methodology ensures that the identified clusters are not an artefact of a single random initialisation but are reproducible and stable. The identification of ‘OC‐related’ and ‘non‐OC‐related’ clusters is a testament to the power of consensus clustering in revealing the underlying biology of OC.

The use of a diverse array of machine learning algorithms to develop and validate prognostic models is a strategic approach to handling high‐dimensional data and feature selection. The random survival forests (RSF), elastic net (Enet) and Lasso regression are particularly noteworthy for their ability to handle such complex data. The calculation of risk scores and the use of Sankey diagrams for visualising risk cluster connections provide a clear and interpretable output that can be directly applied in clinical settings. Gene ontology (GO) classification and KEGG pathway enrichment analysis provide a functional context to the differentially expressed genes, shedding light on the biological processes and pathways dysregulated in OC. The use of CIBERSORT and ESTIMATE algorithms to quantify immune cell infiltration adds another layer to the understanding of OC's tumour microenvironment, which is crucial for immunotherapy strategies.

While our study incorporates single‐cell RNA sequencing (scRNA‐seq) data, we have not fully leveraged this technology's exceptional potential to dissect the heterogeneity of immune cell subpopulations within the tumour microenvironment. This represents a significant missed opportunity for deeper insights into oral cancer immunobiology. The current analysis provides a broad characterisation of major immune cell types but falls short of exploring the nuanced diversity within these populations. For instance, T cells encompass various functional subsets (effector, memory, regulatory, exhausted) with distinct roles in antitumour immunity. Similarly, macrophages exhibit a spectrum of polarisation states from proinflammatory (M1) to immunosuppressive (M2) phenotypes that differently influence tumour progression.

The preprocessing and normalisation techniques applied to scRNA‐seq data using the ‘Seurat’ R package are standard practices that ensure data quality and comparability. The unsupervised clustering analysis and the use of PCA and t‐SNE for dimensionality reduction and visualisation are powerful tools for deciphering the complex landscape of OC at the single‐cell level. The ‘SingleR’ package and ‘FindAllMarkers’ package further enhance the annotation of cell types and the identification of marker genes respectively. When comparing the new model with existing clinical prognostic biomarkers, although the model in this study has shown excellent performance across multiple metrics, the widespread use and maturity of existing clinical biomarkers should not be overlooked. For example, some traditional clinical biomarkers may have been extensively validated over a long period in specific clinical settings, demonstrating high credibility and interpretability. Therefore, the new model needs to undergo more in‐depth comparative studies with these traditional biomarkers in real clinical environments to demonstrate its advantages and complementary value in different clinical scenarios. To better facilitate the transition of the model from research to clinical application, future studies could consider the following directions: First, conduct multicentre, large‐scale real‐world studies to verify the model's universality and stability across different populations and healthcare settings. Second, further optimise the model's interpretability to provide clinical practitioners with more intuitive decision‐making support. Third, explore the combined use of the model with existing clinical biomarkers to achieve more precise individualised prognostic assessments.

## 5 Limitations

Our study is primarily based on computational analyses without experimental validation, which represents a significant limitation. While our machine learning approach has identified potential immune‐related prognostic markers and developed a predictive model for oral cancer, these findings require experimental verification to establish their biological relevance and clinical utility. The lack of in vitro and in vivo validation experiments limits our ability to confirm the functional roles of the identified key genes in immune regulation. Another significant limitation of this study is that the survival analysis did not adequately consider the impact of different treatment modalities received by patients. While this research primarily focused on building a prognostic model based on immune infiltration, it did not conduct stratified analyses of patients under different treatment contexts (such as surgery, radiotherapy, chemotherapy, immunotherapy or combinations thereof). The diversity of treatment regimens can significantly influence patient prognosis, thereby affecting the predictive accuracy of the model.

## 6 Conclusion

This integrative genomic analysis and machine learning approach has uncovered novel OC‐associated genes and provided insights into the immune landscape of OC. The developed prognostic models have the potential to stratify patients into risk groups, which could guide personalised treatment strategies. The findings from this study underscore the importance of a multiomics and machine learning‐driven approach in unravelling the complexity of OC and could serve as a blueprint for future research in this field.

## Author Contributions

**Ling Yang:** conceptualization (equal), data curation (equal), writing – original draft (equal). **Lijuan Guo:** data curation (equal), formal analysis (equal), validation (equal). **Yun Zhu:** investigation (equal), visualization (equal). **Zehan Zhang:** project administration (equal), writing – review and editing (equal).

## Conflicts of Interest

The authors declare no conflicts of interest.

## Data Availability

The data supporting this study can be obtained from the corresponding author upon request.
